# Exploring the Pain-Relieving Potential: Unveiling Antinociceptive Properties in Animal Venoms and Toxins

**DOI:** 10.3390/toxins18020069

**Published:** 2026-01-27

**Authors:** Davi Gomes Angstmam, Bruna Cristina Jeronimo, Joeliton dos Santos Cavalcante, Ana Flávia Marques Pereira, Cristiane Flora Villarreal, Daniel Carvalho Pimenta, Rui Seabra Ferreira Junior

**Affiliations:** 1 Botucatu Medical School (FMB), São Paulo State University (UNESP—Universidade Estadual Paulista), Botucatu 18618-687, SP, Brazil; davi.angstmam@unesp.br (D.G.A.); bruna.jer@gmail.com (B.C.J.); joeliton.cavalcante@unesp.br (J.d.S.C.); ana.f.pereira@unesp.br (A.F.M.P.); 2 Center for the Study of Venoms and Venomous Animals (CEVAP), São Paulo State University (UNESP—Universidade Estadual Paulista), Botucatu 18610-307, SP, Brazil; 3 Laboratory of Pharmacology and Therapeutic (LAFTE), University Federal of Bahia (UFBA), Salvador 40170-290, BA, Brazil; cfv@ufba.br; 4 Laboratório de Ecologia e Evolução, Butantan Institute, São Paulo 05503-900, SP, Brazil; dcpimenta@butantan.gov.br; 5 Center for Translational Science and Development of Biopharmaceuticals FAPESP/CEVAP, São Paulo State University (UNESP—Universidade Estadual Paulista), Botucatu 18610-307, SP, Brazil

**Keywords:** antinociceptive, toxins, nociceptors, therapeutic molecules, pain

## Abstract

Currently, commercially available pain medications can cause adverse effects. Within this framework, researchers have been exploring new drug candidates derived from animal venoms and toxins. The objective of this study was to investigate the number of molecules with potential for pain relief derived from animal venoms and toxins, which could potentially contribute to the development of new biopharmaceuticals. We conducted a literature search in January 2025, covering the period from 1960 to 2025, in two Latin American and nine international scientific databases. The results consisted of 212 articles selected for review. From these articles, 152 toxins and venoms with analgesic potential were identified and classified into 14 different types of pharmacological targets. The peptides investigated, with masses between 500 Da and 5000 Da, are strong candidates for alternative biopharmaceuticals. Most of the toxins found interact with ion channels, representing an alternative to commercially available drugs.

## 1. Introduction

**Figure 1:**
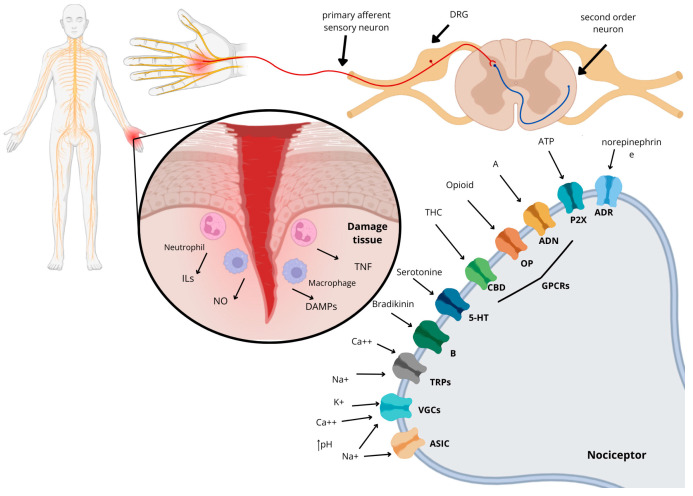
Mechanism of action of pain from a tissue injury that leads to the release of inflammatory factors such as ILs, nitric oxide (NO), TNF, and DAMPs (damage-associated molecular patterns). This process involves the receptors and ion channels that are associated with the nociceptor of the primary afferent sensory neuron, which respond to the nociceptive stimulus. The receptors mentioned are the same as those found in this review: ASICs (acid-sensitive ion channels), VGCs (voltage-gated channels), TRPs (transient receptor potential), B (bradykinin receptor), 5-HT (serotonin receptor), CBD (cannabinoid receptor), OP (opioid receptor), ADN (adenosine receptor), P2X (purinergic receptor), and ADR (adrenergic receptor).

The somatosensory system is part of the physiological process of pain, which is defined as the sensation associated with actual or potential tissue damage. It is always considered a subjective feeling, which encompasses not only physical stimuli but also cognitive and psychological ones, varying from individual to individual and from species to species (Figure 1) [1].

Pain is one of the most frequently encountered symptoms in medical care, especially when it comes to muscle pain [2]. However, it can also be associated with cancer, metabolic and autoimmune diseases, nutritional disorders, traumatic and post-surgical injuries, age, sex, chemical dependencies, sedentary lifestyle, emotional and psychological disorders, social factors, and demographic variations. These are some of the factors that contribute to the development and exacerbation of the clinical picture of pain [3,4].

There are many different types of pain; one of the main methods of classifying pain is according to its duration. Thus, pain can be divided primarily into acute pain and chronic pain [5]. Acute pain is short-term pain that is triggered after a process of injury or tissue trauma, and its prevalence time is between three and six months [6]. Chronic pain is considered a disease in addition to being a symptom of conditions like cancer, arthritis, or autoimmune diseases; it is defined as long-lasting or intermittent pain that persists for at least 3 months [5,7]. Studies on the prevalence of chronic pain in different countries indicate a variation between 9% and 50%. This range is affected by the social and socioeconomic factors of each population and is more noticeable in developing countries, where the patients are mostly women from low-income families, who have a worsening of this disease compared to people from higher social classes [8,9,10,11].

The most commonly used approach to treat pain includes the administration of analgesic medications [12]. However, the majority of medications marketed for pain treatment come with a series of adverse effects, including dependence, tolerance, and constipation. The latter condition often requires an increased dosage of the drug over time due to reduced efficacy and the return of clinical symptoms [13,14,15,16]. Another growing problem in recent years, which has been occurring in several countries, such as the United States, is the presence of dependence caused by the inappropriate use of opioid drugs, especially fentanyl, resulting from a lack of understanding about the adverse effects. The United States has already experienced several opioid crises in which thousands of people died due to dependence on some type of drug. This has led to the need to develop public and social policies to treat this epidemic [17,18,19,20].

One of the alternatives for the development of new drugs is investment in the study of toxinology with animal venom. Venoms are a complex mixture of several molecules [21]. Researchers have come up with better ways to separate, clean, and study molecules derived from animals. This has made it possible to study how the molecules of venomous animals act in the envenomation process and to determine what pharmaceutical and pharmacological properties they might have [22]. These new technologies have enabled the production of new analgesic biopharmaceuticals, such as Prialt^®^, which is derived from the cone snail of the species *Conus magus* and has been the subject of several clinical studies mainly focusing on the treatment of chronic pain [23,24]. Another example is Halneuron^®^, a biopharmaceutical that is composed of tetrodotoxin, a peptide isolated from puffer fish, which is currently in phase II clinical studies [25,26,27]. In addition, there are many other compounds that are undergoing clinical trials or are already on the market [22].

Therefore, the main objective of this study was to present the antinociceptive molecules found in different animal groups, as well as their different mechanisms of action, and to identify those with the potential to be studied as future biopharmaceuticals.

## 2. Results

**Figure 2:**
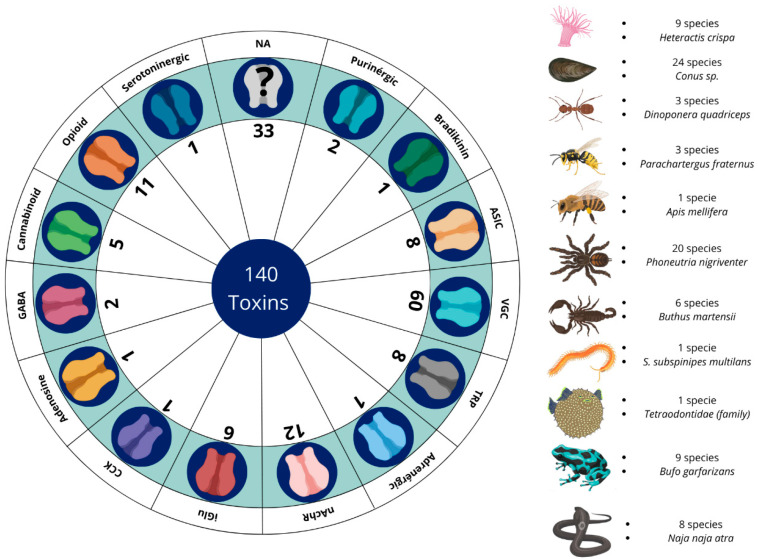
Summary of the quantities of venoms and toxins with antinociceptive potential across their respective receptors and channels, together with the number of species identified by each animal group and which species were the most prominent in the studies. VGC (voltage-gated channel); TRPs (transient receptor potential); ASIC (acid-sensing ion channel); iGluRs (ionotropic glutamate receptors); nAChRs (nicotinic receptors); GABA (γ-aminobutyric acid); CCK (cholecystokinin); NA (not available).

A total of 21,119 articles were found via data collection in databases. After the removal of 4460 duplicates, there were 16,659 articles remaining. During the review of titles, abstracts, and conclusions, it was found that 16,122 articles did not align with the scope of the study and were categorized according to Figure 2 into the following categories: venom characterization, clinical manifestations, envenoming, antimicrobial/parasite, plants, other therapeutic properties, theses, books, congress, and evolution/genetics. Consequently, 537 articles were chosen for full-text reading and included in the qualitative literature study. Following a full-text assessment, 212 articles were selected for the completion of this review. Once the text was read, 152 toxins and venoms with antinociceptive potential were found, separated into 14 different types of ion channel/receptor targets (Figure 2).

### 2.1. Voltage-Gated Channels (VGCs)

Currently, voltage-gated ion channels (VGCs) have been the subject of extensive studies for the purpose of therapeutic innovations aimed at the treatment of chronic and neuropathic pain, tumors, and neurological diseases (Table S1) [28,29,30,31]. These channels are cationic in nature and are subdivided into families, with the sodium ion family having 10 members (1.1–1.9 Nav), potassium channels (1 to 12 Kv members), and calcium ion channels (1.1–1.4, 2.1–2.3, and 3.1–3.3 Cav). Each member of this family is associated with a specific type of nociception and a distinct region of the body with wide distribution in the central and peripheral nervous system. They are present in the membrane of sensory neurons and are activated by changes in the electrical potential near the channels, as well as by the presence of salts in the extracellular medium. This means that the transmission of the sensation of nociception is directly related to the excitability and inhibition of these channels [32,33,34,35,36]. *Conus* species are widely studied for producing toxins that interact with VGCs; among them, ω-conotoxin MVIIA stands out. Popularly known as ziconotide, this peptide is the main component of the drug Prialt^®^ [23]. It interacts with Cav2.2, leading to its inhibition, which causes an analgesic effect on the body. In models of neuropathic pain caused by nerve injury, chronic pain, postoperative pain, and visceral pain, this molecule showed antinociceptive effects when administered intracerebroventricularly, intranasally, intrathecally, and subcutaneously [37,38,39]. Despite these promising results, scientists have observed that this toxin can cause motor dysfunction.

The transition from parenteral administration to non-invasive routes faces significant challenges, including proteolytic and acid degradation in the gastrointestinal tract, low epithelial permeability due to the hydrophilic nature of peptides, and the restriction imposed by the blood–brain barrier. To mitigate these limitations, the use of nanocarriers, such as liposomes and micelles, has emerged as a promising strategy to increase the metabolic stability and bioavailability of these molecules. Recently, researchers associated this peptide with micelles, and the results demonstrated that the toxin maintained its antinociceptive action without causing adverse effects [39,40]. When compared to opioid drugs, ω-conotoxin MVIIA showed antinociceptive potency about 800 times higher than morphine without inducing pharmacological tolerance. Furthermore, this molecule also potentiates the effect of morphine [41,42].

Other toxins from *Conus* sp. also exhibit important antinociceptive properties. The toxin Bu8, *C. bullatus*, inhibits Ba^2+^ currents in Cav channels, exhibiting an antinociceptive effect twice as high as that of ω-conotoxin MVIIA in models of acute and inflammatory pain induced by acetic acid, presenting low motor dysfunction and toxicity in fish [43]. On the other hand, AM336 (*C. catus*) showed a dose-dependent antinociceptive effect intrathecally in models of inflammatory pain caused by complete Freund’s adjuvant (CFA) without the manifestation of side effects [44]. The toxin KIIIA derived from *C. kinoshitai* blocks Nav channels, producing an antinociceptive effect in formalin models when administered intraperitoneally; the tests were performed together with some synthetic analogs, which demonstrated promise for the development of new analgesic drugs [45,46]. The toxin MrVIB, derived from *C. marmoreus*, inhibits persistent pain behavior by blocking the propagation of action potentials in Nav1.8 channels intrathecally in models of chronic constriction injury. However, in high doses, it can lead to motor problems [47,48,49].

Also, the toxins MoVIA (*C. moncuri*), RsXXIVA (*C. regularis*), and SO-3 (*C. striatus*) block Cav 2.2, thereby reversing pain behavior in neuropathic pain models resulting from nerve damage and inflammatory pain induced by formalin and acetic acid. The toxin TsIIIA from *C. tesselatus* also blocks Nav 1.8, which has a long-lasting effect on lowering pain and raising the nociceptive threshold [50,51,52,53,54].

In toxin-producing spiders, a total of 20 species that interact with these receptors have been identified, of which *Phoneutria nigriventer* stands out, presenting the largest quantity of toxins, namely Phα1β, Pntx3-3, and PhTx3-4, which act by inhibiting Cav channels. Phα1β is one of the most extensively studied toxins. In inflammatory pain experiments using the molecule and morphine, the standard drug demonstrated adverse effects such as increased hyperalgesia, while Phα1β reversed these effects [55]. Phα1β also reduced nociception to heat and decreased gastrointestinal transit; experiments with the recombinant molecule presented effects similar to those of the native molecule. It was also demonstrated that the co-administration of Phα1β with morphine potentiated the antinociceptive effect of the drug’s analgesia. However, Phα1β presented motor impairment in mobility tests such as the rotarod [56,57,58,59]. In models of cancer pain induced by melanoma cells in the hind paw of mice, Phα1β caused the reversal of pain hypersensitivity and, compared with ω-conotoxin MVIIA, did not demonstrate adverse effects [60,61,62]. Administration of the Phα1β peptide resulted in the reversal of inflammatory pathology in peripheral glial cells caused by CFA through inhibition of Cav channels, generating an antinociceptive response to inflammatory pain [63].

Pntx3-3, on the other hand, did not reduce inflammatory pain, manifesting action only in models of nociceptive and neuropathic pain (diabetic neuropathy and partial injury to the sciatic nerve), and demonstrated a pain-reducing effect in models of fibromyalgia induced in mice [64,65]. Pntx3-4 may be related to capsaicin-induced glutamate inhibition in a calcium-dependent and independent manner, which may suggest a possible action on NMDA (N-methyl-D-aspartate) receptors. It also did not cause changes in locomotor activity. In tests with formalin, it did not demonstrate an acute nociceptive effect, only showing efficacy in the late phase of the test. The toxin caused an improvement in mechanical hypersensitivity and, in high doses, led to paralysis and weakness [66,67].

Within the genus *Cyriopagopus* sp., two species are responsible for the production of toxins Ca1a, Ca2a, and Cyriotoxin-1a. These toxins strongly block Nav 1.7 and 1.6, which stops pain responses to formalin and acetic acid and lowers abdominal writhing and thermal nociception [68,69,70]. The toxins Hntx-III and -IV, derived from the species *Ornithoctonus hainana*, exhibit inhibitory action in Nav 1.7, 1.2, and 1.3, causing a reversal of inflammatory and neuropathic pain; a reduction in abdominal writhing, acute pain, and hyperalgesia; and inhibition of allodynia without motor impairment. However, in high doses, they caused paralysis in mice [71,72,73,74]. Studies involving *Agelenopsis aperta* have identified a Cav channel-blocking toxin that demonstrated antinociceptive potential in rat models of carrageenan-induced inflammatory knee pain when administered topically [75,76].

The spider, *Ceratogyrus darlini*, produces a toxin called Cd1a that interacts with Cav2.2 and Nav channels in electrophysiological tests and in models of pain caused by the scorpion toxin OD1 [77]. The species *Chilobranchys jingzhao* produces the toxin jingzhaotoxin-34, which acts selectively on Nav 1.7 channels and has also demonstrated a more potent and long-lasting effect than morphine in tests of inflammatory pain caused by formalin, especially in the late phase [78]. Phlotoxin-1, derived from *Phlogiellus* sp., reduces the nociceptive response to formalin in both phase 1 and phase 2, as well as to the scorpion toxin OD1 [79,80]. Protx II/III toxins from *Thrixopelma pruriens* inhibit Nav1.7 channels, reversing pain induced by OD1 and dorsal root ganglion (DRG) injury [81,82]. *Davus fasciatus* produces the toxin Drtx-Df, which inhibits Nav1.7 and Cav3 channels, thereby reversing pain behavior induced by OD1 [83].

*Grammostola rosea* produces the toxins Gptx1 and Gptx1-71 with excellent selectivity for blocking Nav1.7. Both have antihyperalgesic effects on inflammatory pain via intrathecal and intraplantar routes in a dose-dependent manner, manifested by the action of CFA and OD1. The effects were attenuated by opioid antagonists, indicating the possible participation of the toxin in the endogenous opioid system. Furthermore, it also induced spinal analgesia without losing potency over 8 days in models of neuropathic pain and inflammation [84,85,86].

The species *Haplopelma lividum* secretes the toxin H1a, which acts by inhibiting Nav1.8 channels. It was able to dose-dependently reduce inflammatory and neuropathic pain in formalin, acetic acid, and hot-plate tests, showing a better effect than morphine [87]. The neurotoxin HpTx3, derived from the venom of the huntsman spider, *Heteropoda venatoria*, has a potent inhibitory effect on Nav 1.7 channels; this activity is dose-dependent and was observed in the following pain models: those induced by formalin, acetic acid, CFA, the hot plate, and nerve injury [88].

Within the spider group, it has been demonstrated that huwentoxins I, IV, and XVI, extracted from *Ornithoctonus huwena*, block both Cav and Nav channels, resulting in an antinociceptive effect comparable to that of ω-conotoxin MVIIA and morphine. Tests involving inflammatory pain, formalin-induced allodynia, OD1, and incisional injury demonstrated this effect without presenting motor dysfunction [89,90,91]. SNX-482, a toxin from *Hysterocrates gigas* that acts on Cav channels (specifically Cav2.3), was administered intravenously and shown to reduce pain in models of neuropathic pain caused by spinal nerve ligation injury and mechanical allodynia [92].

The scorpion genus *Buthus* sp. is the group with the second-highest quantity of molecules with action on VGCs, totaling 15 toxins. These toxins induce pain reduction in models of visceral pain, inflammatory pain, and chronic sciatic nerve constriction [93,94,95,96,97,98,99,100,101,102,103,104,105,106,107,108,109,110,111,112]. The scorpion species *Hottentotta franzwerneri* produces the toxin ω-Buthitoxin-Hf1a, capable of inhibiting calcium influx via Cav3.3 and Cav3.2 channels, producing an antinociceptive effect in mechanical and thermal allodynia caused by the post-surgical pain model [113]. The venom of the scorpion *Heterometrus laoticus* has a high affinity for Kv1.3 channels. Researchers of one study administered a solution containing the crude venom into animals under study, subjecting them to acetic acid writhing and carrageenan-induced paw edema tests. The crude venom induced an important antinociceptive and anti-inflammatory action [114].

Gln49-PLA2 is a phospholipase A2 that is found in the venom of the snake *Gloydius ussuriensis*. It raised the threshold of thermal pain in mice in a dose-dependent way, even though it was not lethal. This action was antagonized by naloxone, indicating the possible mediation of opioids in the action of the toxin. However, electrophysiological studies have shown that the main action of this toxin is in the depolarization of Kv channels [115,116].

Tetrodotoxin was first described in the Tetraodontidae fish family [117]. It is a toxin that interacts with Nav channels and has played a significant role as a subcutaneous or topical antinociceptive and local anesthetic in models of inflammatory, neuropathic, and oncological pain. It was able to reduce allodynia and hyperalgesia manifested by carrageenan, formalin, paclitaxel, and DRG injury [118,119,120,121,122,123,124]. The toxin is undergoing clinical studies as part of the drug Halneuron [26].

Other groups of animals, such as amphibians represented by *Trachycephalus typhonius* and *Bufo gargarizans*, produce the toxins Tt7 and bufalin, respectively. These toxins inhibit Nav, while bufalin causes the suppression of the inflammatory pain response caused by carrageenan [125]. Tt7 lowered pain caused by heat in the hot-plate test and stopped calcium from working on DRG neurons, which suggests it may be working on ASIC receptors [126]. The skin of the tree frog, *Hyla annectans*, releases a peptide called anntoxin. This peptide blocks Nav channels and has been shown to be very effective at relieving pain in a number of models, including the tail-flick test, the hot-plate test, the acetic acid-induced writhing test, the formalin-induced paw licking test, and the carrageenan-induced paw edema test [127].

The venom of the snake *Naja atra* was found to block Nav1.8 channels in models of inflammatory and neuropathic pain without demonstrating toxicity and with an effect more potent than that of morphine [128]. Additionally, the centipede species *Scolopendra subspinipes mutilans* produces the toxin SSm6a, which blocks Nav1.7 and works better than morphine in models of pain caused by acetic acid and formalin [129].

### 2.2. Opioid and Cannabinoid Receptors

Opioid receptors are responsible for the pharmacological effects of opioid antinociceptives, the class considered the gold standard for antinociceptive studies. They are located in central and peripheral neurons, as well as in certain cell types, such as endocrine, immune, and dermal cells. In mammals, they are divided into three types: mu (µ), delta (δ), and kappa (κ) [130,131,132]. In addition to the receptors, there is also the endogenous opioid system, which is distributed throughout both the central and peripheral nervous systems and is composed of three classes of peptides called endorphins, enkephalins, and dynorphins. This system performs several functions in the body, including analgesia and mood modulation (Table S2) [133,134,135].

Adolapine is a peptide isolated from bee venom. This molecule consists of 103 amino acids and has a molecular weight of 11 kDa. It demonstrates a potent antinociceptive effect by inhibiting the activity of prostaglandins. Studies indicate that adolapine induces antinociception, a process modulated by opioid receptors, as evidenced by the inhibition of its effects upon administration of opioid receptor antagonists [136].

Some of the toxins discovered in amphibians are known as “Kambô,” a term referring to the venom of frogs that Indigenous Amazonian peoples have historically used as a form of medicine for treating infections, preventing diseases, and as an antinociceptive. Initially researched in the frog species *Phyllomedusa bicolor*, these medical studies have expanded to encompass various other amphibian species [137]. Among the molecules with antinociceptive properties found in amphibians, three main groups stand out: caeruleins, dermorphins, and deltorphins [138,139].

Dermorphins are heptapeptides with an affinity for µ-type opioid receptors. In preclinical tests, they have demonstrated longer-lasting and more potent effects than morphine, with up to 1000 times greater efficiency when administered intrathecally. Dermorphins are found in the skin of amphibians of the genus *Phyllomedusa* sp. [140,141]. Deltorphins are also heptapeptides that bind to β-opioid receptors. However, they work a little differently than dermorphins because they depend on glial cells [142,143].

Hannalgesin from the venom of the king cobra (*Ophiophagus hannah*) is a 72-amino acid neurotoxin exhibiting opioid characteristics. It exhibited an antinociceptive effect even when administered orally, and studies have indicated that the antinociceptive effect of the molecule is related to the presence of nitric oxide. However, depending on the dose administered, it can cause neurological deficits, shrinkage of muscle membranes, and darkening of mitochondria in skeletal muscles [144].

Another snake species known to possess molecules with antinociceptive properties in its venom is the rattlesnake (*Crotalus durissus terrificus*). The first studies with this species’s crude venom showed that it could relieve pain through the mouth, the bloodstream, the peritoneum, and the skin. These effects were caused by opioid receptors.

Additionally, the active component was identified as a low-mass molecule [145,146]. In 1998, research with a neurotoxin that was about 4.8 kDa showed that it could reduce pain through opioid receptors. Remarkably, this toxin exhibited potency up to 500 times greater than morphine at molar doses while causing no apparent tissue damage in mice. Consequently, it was distinguished as the unique low-molecular-weight antinociceptive molecule identified at that time [146]. Subsequently, investigations were conducted to elucidate the venom’s interaction with opioid receptors and to determine the involvement of nitric oxide and potassium channels in its antinociceptive properties. These experiments were performed using models of hyperalgesia induced by prostaglandin E2 and carrageenan [147,148,149].

In 2003, studies on *C. d. terrificus* crotamine revealed that, despite behaving similarly to defensins and sharing similar structures, the molecule could also interact with Nav channels [150]. Subsequently, in 2021, a study using recombinant crotamine molecules showed biological activity, pointing to a possible pain-relieving effect through opioid receptors [151,152].

In relation to rattlesnake venom, other bioactive molecules have also been discovered and continue to be studied to this day. In 2008, a low-mass molecule consisting of around 15 amino acids was identified, known as crotalphin, which is a γ chain of crotapotin. Crotalphin exhibited antinociceptive effects via various routes of administration, including oral, intravascular, subcutaneous, intraplantar, intrathecal, and peritoneal, with an efficacy approximately 10 times greater than morphine, particularly in peripheral analgesia [153].

Furthermore, crotalphin showed promising results in models of neuropathic pain induced in the sciatic nerves of mice, demonstrating sustained antinociceptive effects. It exhibited interactions with both opioid and cannabinoid receptors, making it a potential candidate for chronic pain treatment. Crotalphin also worked well in tests that measure inflammatory pain, like the formalin and acetic acid tests, and it was also effective in treating cancer pain [153,154,155].

In the snake *Naja n. atra*, a molecule called najanalgesin has been discovered that acts on opioid receptors, demonstrating antinociceptive effects with greater efficacy when administered intrathecally and intraperitoneally. Furthermore, there is a significant association with astrocytes, showing positive results in studies involving induced neuropathic pain [156,157]. In studies with the species *Micrurus lemniscatus*, antinociceptive properties targeting opioid receptors have been identified. However, little is currently known about the specific toxin responsible for this effect [158].

Cannabinoid receptors have been discovered through studies on the medicinal properties of *Cannabis* sp., which play an important role in immunomodulation. To date, two types of receptors have been discovered—CB1 and CB2—both associated with G proteins [159]. CB1 receptors can be found mainly in the central and peripheral nervous systems, skin, and liver, while CB2 receptors act mainly in the peripheral nervous system and in cells of the immune system [159,160,161]. Both are arranged in vesicles of nerve cells in mammals, mainly in glial cells, divided between the central and peripheral nervous systems, respectively. They have a broad affinity with lipid molecules and can inhibit mechanical, thermal, and chemical nociceptive effects, in addition to being related to the treatment of neuropathic and chronic pain [162,163].

In a study by Jergova et al., the effects of *Conus* venom on cannabinoid receptors were investigated. For this purpose, HEK293 cells expressing CB1 and CB2 cannabinoid receptors were used to select the fractions with the greatest antinociceptive potential. These selected fractions underwent additional fractionation steps and were tested in experimental pain models. Among the species evaluated, *C. textile* demonstrated significant antinociceptive effects in the formalin test, in addition to attenuation of thermal and mechanical allodynia in a nerve injury model [164].

PnPP-19 and ε-Ctenitoxin-Pn1a, two toxins found in the spider *P. nigriventer*, were purified and used to study biological processes connected with pain. Experiments using mechanical, inflammatory, neuropathic, and acute pain models revealed that these toxins exhibit effects on opioid and cannabinoid receptors [165,166,167].

In the case of anuran toxins, it is noteworthy that not all identified toxins are of protein origin; some molecules are organic compounds. For example, bufalin, which is synthesized from the toxins of *B. gargarizans*, reduces inflammation by blocking anti-inflammatory substances. Furthermore, it interacts with opioid and cannabinoid receptors, thereby alleviating inflammation, inflammatory pain, and cancer-induced pain [168,169].

### 2.3. TRP (Transient Receptor Potential) Channels

TRP channels were initially characterized in studies with the species *Drosophila* sp. [170,171]. TRPs are cation channels expressed by sensory neurons and play important roles in the generation of pain when exposed to physical and chemical stimuli, especially temperature variations, such as the TRPV1 channel, which is sensitive to the extreme heat generated by capsaicin [172,173,174,175]. In some cases, certain subunits of these channels present additional sensitivity to protons, contributing to the peripheral perception of inflammatory pain [172,176]. This group of receptors has seven subfamilies: TRPA, TRPV, TRPP, TRPC, TRPM, TRPML, and TRPN. Each of these subfamilies has six different transmembrane domains based on their amino acid sequences and subunit variations [176,177,178]. Calcium and magnesium ions activate these channels, which are non-selective and preferably thermosensitive, but other stimuli can also activate them (Table S3) [179,180].

TRP modulation has been implicated in the antinociceptive properties of toxins. The sea anemone *Heteractis crispa* exhibited two molecules with action on TRPV1, APHC-1, and APHC-3, both of which consist of 56 amino acid residues and weigh 6 kDa. Electrophysiological experiments have shown that these toxins partially inhibit these receptors. In tests using models of pain and inflammation, an increase in the latency of response to noxious stimuli was observed upon administration of APHC-1 and APHC-3 [181,182].

Another toxin that interacts with this receptor is Ms9a-1, derived from the sea anemone, *Metridium senile*. Using models of pain and inflammation, the authors investigated its action through in vitro and in vivo tests. The data suggests that, although it cannot activate the receptor directly, the molecule acts as a positive modulator by enhancing receptor activation through different agonists. The results were very positive, since the molecule was able to produce antinociceptive effects and inhibit the neurogenic inflammatory response at low doses and without causing side effects [183]. In a recent study, the molecule showed pain-relieving and anti-inflammatory effects in models of monosodium iodoacetate (MIA)-induced osteoarthritis, with results equivalent to or superior to those observed for the reference drugs, meloxicam and ibuprofen [184]. Another species of anemone, *Urticina eques*, produces the toxin Ueq 12-1, which acts on TRPA1 receptors similar to Ms9a-1. It also exhibits antibacterial activity in addition to its antinociceptive properties [185].

The spider *P. nigriventer* has been found to contain two antinociceptive toxins in its venom. One of these is PnTx3-5, which acts on TRPV1 receptors, causing an inhibitory effect. It has demonstrated antinociceptive effects in models of postoperative pain, neuropathic pain, and cancer-induced pain. It has been particularly effective in animals with opioid tolerance [186,187].

Another toxin from *P. nigriventer* called Phα1β has been shown to effectively block TRPV1 receptors in capsaicin models, leading to pain-relieving and anti-allergic effects. In order to assess its efficacy in relation to ω-conotoxin MVIIA, the molecule was tested in peripheral pain models via intradermal and intracerebral injection [188]. Additionally, Phα1β has been shown to relieve pain without any signs of toxicity when compared to ω-conotoxin MVIIA in models of neuropathic pain and cancer, with effects that last for up to 14 days when injected into the spinal cord [189,190]. It has also been shown that this molecule can lower mechanical hyperalgesia, which happens when TRPA1 channels are activated along with Cav channels [191]. In studies that evaluated pain relief after surgery, low doses of Phα1β toxin increased the pain-relieving effects of morphine while also lowering tolerance, withdrawal syndrome, and pain that was caused by opioids. This result suggests that it could be an effective adjuvant when used in combination with opioids for the treatment of postoperative and cancer pain [55,189].

Experiments with the spider toxin GsMTx-4 from *G. rosea* in models of neuropathic and inflammatory pain demonstrated a reduction in mechanical hyperalgesia through TRPV4 receptors, in cooperation with two other members of the TRP channel subfamily, TRPC1 and TRPC6, which are expressed in neurons of the DRG [192]. GsMTx-4 was also produced recombinantly in vitro and retained its antinociceptive activity, alleviating mechanical hyperalgesia and allodynia caused by inflammation and nerve damage [193].

The BmK AGAP toxin, derived from the venom of the scorpion *B. martensii*, has been shown to inhibit TRP receptors. When combined with lidocaine, this molecule can induce long-lasting analgesia, making it suitable for postoperative treatments with local anesthetics administered intrathecally [101,194].

Another toxin that interacts significantly with TRP channels is crotalphine. Experiments on HEK293t kidney cells and dorsal root ganglion cells, both expressing these channels, have demonstrated that crotalphine from *C. d. terrificus* activates the TRPA1 channel, leading to long-lasting desensitization. This mechanism is crucial for the antinociceptive and antihyperalgesic effects observed in C. d. t. venom [195].

### 2.4. ASICs (Acid-Sensing Ion Channels)

ASICs are a class of receptors located on neurons in both the central nervous system (CNS) and the peripheral nervous system (PNS). They are also associated with mechanoreceptors and play a crucial role in stimulus mechanical transduction [196]. These channels are activated by the presence of protons in the synaptic space, leading to a mild acidification of the environment, which causes the channels to open or close [197,198]. There are six different subunits in the ASIC channel family. They are called ASIC1a, ASIC1b, ASIC2a, ASIC2b, ASIC3, and ASIC4 and are encoded by a different set of four genes. These receptors have a notable sensitivity, being able to respond to pH differences as small as 0.2 units while still being able to recognize very minor pH variations (Table S4) [196,197,198,199,200,201].

Protons released into the extracellular environment during the inflammatory process can activate ASICs, contributing to inflammatory pain [202]. This activity may be associated with pain clusters resulting from a variety of conditions, including ischemia, hematoma, fracture, tumor development, and muscle and skin incisions after surgical procedures, and has been linked to diseases such as arthritis, urogenital tract obstructions, and digestive disorders [200]. Some toxins found in cnidarians have also been found to possess antinociceptive properties by selectively targeting ASIC receptors. In particular, these include toxins from the anemones *H. crispa* (Hcr1b-1, -2, -3, and -4) [203,204] and *Urticina grebenyi* (UGR9a-1) [205].

Mambalgins I, II, and III are three neurotoxins with antinociceptive effects found in the venom of the black mamba snake, *Dendroaspis polylepis*. These molecules, each composed of 57 amino acids, differ by only one amino acid from each other [206]. These toxins have demonstrated a strong affinity for ASIC1a, ASIC2a, and ASIC3, acting quickly and reversibly and causing few side effects. Diochot et al. demonstrated that mambalgins are capable of inhibiting ASICs expressed either in central or peripheral neurons. The authors also found that mambalgins and their isoforms showed promising results in treating neuropathic and inflammatory pain, which shows how useful they could be in the field of pain treatment [206,207].

### 2.5. Nicotinic Acetylcholine Receptors (nAChRs)

Nicotinic acetylcholine receptors form ion channels through the junction of their five transmembrane domains, and the main subtypes are α6β4, α7, and α9α10. They are distributed throughout the CNS and PNS. These receptors are involved in different physiological functions, such as cognition, memory, learning, and mood modulation (Table S4) [208,209,210]. When activated, nAChRs play roles in pain modulation by inhibiting the release of inflammatory cytokines, thus producing, in addition to an anti-inflammatory action, an analgesic response in the body, mainly in models of neuropathic pain [211,212].

Epibatidine is an alkaloid toxin found in the amphibian species *Epipedobates tricolor*, which has a similar structure to nicotine and acts as an agonist in nAChRs, with an antinociceptive effect in models of inflammatory and neuropathic pain [213,214,215]. In tests with mice’s spinal cords, epibatidine proved to be a fully selective and potent agonist at these receptors; it also showed tolerance with continuous use [216,217,218].

In the mollusks group, seven toxins (α-conotoxin Lv1d, α-conotoxin BuIA, α-conotoxin RgIA, α-conotoxin Vc1.1, α-conotoxin Mr1.1, lt14a, and αO-conotoxin GeXIVA) from species of the genus *Conus* sp. (*C. lividus*, *C. bullatus*, *C. regius*, *C. victoriae*, *C. marmoreus*, *C. literatus*, and *C. generalis*) were found to interact with nAChRs and have shown antinociceptive effects in models of neuropathic and inflammatory pain [219,220,221,222,223,224,225,226,227]. Specifically, RgIA was able to reduce hypersensitivity in a model of neuropathic pain induced by oxaliplatin [224]. In experiments with synthetic α-conotoxin BuIA, the authors demonstrated positive results, as the molecules were able to attenuate pain in the hot-plate model and the paclitaxel-induced peripheral neuropathy model without causing adverse effects [219].

Various species within the Elapidae snake family possess toxins that specifically target nAChRs. Cobrotoxin, from *Naja n. atra*, is a substance that may induce analgesia in the CNS [228]. Cobratoxin (CTX) isolated from *Naja n. kaouthia* venom has antinociceptive potential in a dose-dependent cancer-induced bone pain model through nicotinic acetylcholine receptors [229]. CTX was also able to inhibit inflammatory pain caused by formalin, pain caused by CFA-induced arthritis, and neuropathic pain caused by disruption of nerve connections and was shown to be opioid-independent [230,231,232,233].

In models of inflammatory pain, the organic compound cinobufagin, obtained from the amphibian *Bufo b. gargarizans*, showed an antinociceptive effect by activating nAChRs [234]. The armored spider *P. nigriventer* also has a toxin in its venom that is capable of modulating pain by activating cholinergic receptors of the muscarinic and nicotinic systems in models of neuropathic and inflammatory pain [235].

### 2.6. γ-Aminobutyric Acid (GABA) Receptors

γ-Aminobutyric acid (GABA) is an inhibitory neurotransmitter responsible for regulating mammalian behavior, such as sensations, consciousness, sleep, learning, and memory, but it is also related to chronic pain conditions. These molecules target GABA receptors, which are widely distributed throughout the CNS in neurons and glial cells. These receptors can be divided into ionotropic (GABAA and GABAC) and metabotropic (GABAB) types, which are coupled to G proteins [12,236,237]. Ionotropic GABA receptors modulate pain when presynaptic receptors, stimulated by the presence of the neurotransmitter, promote the efflux of Cl^−^ from the afferent neuron to the synaptic cleft, leading to influx through the GABA receptor located in the postsynaptic neuron, generating hyperpolarization, and thus generating the sensation of pain [238,239]. In comparative studies, the antinociceptive function of GABA receptors has shown satisfactory results in models of neuropathic, chronic, and inflammatory pain induced by CFA and formalin (Table S4) [240,241,242].

Several in vitro and in vivo experiments have demonstrated that conotoxins modulate the action of Cav channels while also causing the activation of GABAB receptors [243,244,245].

The conotoxins RgIA, GeXIVA, Vc1.1, and AuIB, along with their respective synthetic analogs, have previously shown antinociceptive potential in models of neuropathic pain, mechanical allodynia, and nerve injury by modulating GABAB receptors [224,225,237,246].

### 2.7. Ionotropic Glutamate Receptors (iGluRs)

Ionic glutamate receptors are fundamental elements in the regulation of synaptic transmissions in neurons involved in sensory transmission and cerebral stimulus processing. There are four functional groups of receptors: AMPA (amino-3-hydroxy-5-methyl-4-isoxazolepropionic acid), kainate, NMDA, and GluD (also called delta receptors) (Table S4) [247,248,249].

iGluRs are responsible for triggering long-lasting responses in synapses; they are associated with processes such as neuropathic pain, learning, and memory. In the treatment of neuropathic and chronic pain, when antagonized, glutamate receptors play a significant role in pain relief. These receptors can also change how endogenous opioids are released in the body and how opioid tolerance is built up by exogenous molecules. This may be important for treating chronic pain and controlling pain in general [250,251,252].

In studies with various toxins with anti-inflammatory and antinociceptive potential from *Conus* cone snails, some compounds showed affinity for glutamate receptors [253]. An electrophysiological study conducted with conotoxin-AC1 and its synthetic variants demonstrated that the toxin originating from *C. achatinus* was capable of inhibiting NMDA receptors in HEK293 cells. In murine pain models, the administration of the molecule resulted in dose-dependent analgesia [254]. The species *C. geographus* has two conantokins, G and T, and their synthetic variants have an affinity for subtypes of NMDA receptors, which exhibit antinociception in an injury-induced pain model [255,256].

Joro spider toxin (JST), obtained from the venom of *Trichonephila clavata*, can interact with glutamate receptors, specifically the AMPA type. In experiments with pain models, the molecule was able to inhibit the receptor, causing analgesia in models of thermal hyperalgesia and mechanical allodynia pain, but it did not produce results in the inflammatory pain model induced by formalin [257,258].

The *P. nigriventer* peptide Pntx(5-5) showed an antinociceptive effect in the inflammatory phase of the formalin test by inhibiting NMDAR receptors. This peptide is capable of mediating the antinociceptive effect in the spinal cord, not only on postsynaptic receptors but also through possible interactions with autoreceptors [259].

The Brazilian scorpion *Tityus serrulatus* produces a toxin called TsNTxP. This toxin may be able to block NMDA receptors by decreasing the amount of glutamate in the body during fluorescence experiments with these receptors. Furthermore, the molecule demonstrated antinociceptive effects in models of allodynia and neuropathic pain induced by CCI and paclitaxel [260].

### 2.8. Purinergic Receptors

Purinergic receptors are a family of receptors that can be divided into two main types: P1 and P2. P2Y receptors are ATP receptors coupled to G proteins, while the P2X class is also mediated by ATP but is permeable to Na^+^, K^+^, and Ca^2+^ cations (Table S4) [261,262,263].

Activation of these receptors is primarily related to controlled ATP release into the environment, allowing the influx of hypertension and calcium into the cell, and has been associated with various types of pain, including neuropathic pain, cancer pain, allodynia, and acute inflammatory pain [262,264,265]. *Geolycosa* sp., a genus of spider, was found to produce two toxins, purotoxin-1 and purotoxin-2, that interact with purinergic receptors. Both block P2X3, which lowers the affinity between the agonist and the receptor. This effectively reduces pain in models of inflammatory pain [266,267].

The *B. gargarizans* bufalin molecule also showed interaction with P2X7-type receptors, inhibiting the channel’s activity and regulating the release of inflammatory cytokines, such as TNF-α, IL-18, IL-1β, and IL-6 in models of neuropathic pain [268].

### 2.9. Adrenergic Receptors

Adrenergic receptors located in the spinal cord are involved in inflammatory and neuropathic pain [269,270]. These receptors are activated by drugs such as clonidine and dexmedetomidine and are associated with the control of chronic pain (Table S4) [269,271,272].

The bee *Apis mellifera* venom has been extensively studied as an adjuvant for pain treatment. In several studies, melittin, one of the venom’s toxins, produced an antinociceptive effect via adrenergic receptors in models of paclitaxel-induced neuropathic pain, osteoarthritis, and visceral pain induced by acetic acid [273,274,275,276]. The use of diluted bee venom (DVB) at acupuncture points, a technique known as apipuncture, is an established practice in Eastern medicine for the therapeutic management of various pathological conditions. In an experimental study, the antinociceptive effects of this approach were evaluated in models of neuropathic pain. The results indicated that DVB exerts its effect through the activation of alpha2-adrenoceptors and that the administration of the compound twice daily resulted in a significant reduction in thermal hyperalgesia and mechanical allodynia in the animals tested [276,277].

### 2.10. Bradykinin Receptors

Bradykinin receptors are a family of G protein-coupled receptors characterized by having seven transmembrane domains. These receptors can be classified into two main groups, known as B1 and B2, and are distributed in tissues such as endothelial, cardiovascular, respiratory, and immune cells, exhibiting different physiological functions [278,279,280]. Bradykinin interacts with receptors, causing excitability in afferent pain receptors and hyperalgesia, in addition to causing the production of inflammatory mediators. Studies with antagonists of the bradykinin B2 receptor showed that inhibiting the receptors generated positive responses for antinociception in models of inflammatory (CFA) and neuropathic (CCI) hyperalgesia [281]. In wasps, the species *Polybia occidentalis* presents a peptide analogous to Thr6-bradykinin, which shows affinity for the B2 receptor subtype, causing a dose-dependent thermal antinociceptive effect and demonstrating that it is more potent than morphine and bradykinin (Table S4) [282].

### 2.11. Adenosine Receptors

Adenosine interacts with a family of G-protein-associated receptors, which are distributed in different tissues according to their subtypes (A1R, A2AR, A2BR, and A3R) [283,284]. These receptors play multiple physiological roles, including the regulation of cardiovascular, respiratory, renal, inflammatory, and immunological processes [285]. In pain, adenosine receptors, when activated, promote the inhibition of the action of cAMP, protein kinase A, and calcium channels, leading to reduced pain perception in models of chronic and inflammatory pain induced by formalin and carrageenan [286,287,288]. It has been shown that the venom of the *Naja n. atra* snake can reduce pain by activating adenosine receptors, especially those in subtypes A1 and A2a. In moderate doses, these effects result in antinociception, while high doses can trigger hyperalgesia. Activation of subtype A1 is associated with antinociception, whereas activation of subtype A2a, at higher concentrations of the neurotoxin, can cause hyperalgesia (Table S4) [289].

### 2.12. Cholecystokinin Receptors (CCKr)

Cholecystokinin receptors are a class of receptors composed of two main subclasses (CCK-A and CCK-B) distributed mainly throughout the CNS and enteric nervous system (ENS). They are associated with sensitivity, anxiety, and motility of the gastrointestinal system [290,291]. The DRG in the peripheral system and the spinal cord in the CNS both contain this class of receptors, which stimulate the release of neurotransmitters associated with pain, such as glutamate and GABA. The activation of CCKr leads to hyperalgesia and inflammatory pain [292,293,294,295]. CCKr may act to reduce tolerance triggered by opioid molecules (Table S4) [295].

Caeruleins are composed of 10 to 16 peptides and are known for their antinociceptive properties. They were first isolated in the frog *Hyla caerulea* but have subsequently been found in several other genres [296,297]. They are known as myotropic peptides with contractile effects on the intestines and smooth muscles. These endogenous peptides are significant in the skin of amphibians and have a structure similar to gastrin in mammals and cholecystokinin (CCK). Caeruleins can act through CCKr in the digestive tract, causing constriction and slowing down gastric emptying in response to protein and fat intake [138,294]. Caeruleins are not opioid peptides and have been shown to be 15 times more effective than morphine. Their antinociceptive properties have been evaluated in various indications in oncological, gastrointestinal, and migraine domains [298,299]. Recent years have revived studies with caeruleins, primarily due to their potential in oncological treatment through intrathecal administration into the spinal cord [138].

### 2.13. Serotonin Receptors

Serotonin (5-HT) is a neurotransmitter that acts on G-protein-coupled receptors that, in addition to being related to mood modulation, play a fundamental role in pain modulation [239]. Serotonin receptors are divided into six families, of which only 5-HT1A, 5-HT3, and 5-HT7 play a role in pain reduction when activated [300,301,302,303]. Only the peptide named bunodosine 391, an acid acylamine from the anemone *Bunodosoma cangicum,* showed an antinociceptive effect in carrageenan-induced hyperalgesia. Bunodosine does not demonstrate an antinociceptive effect blocked by naloxone; however, its effect was reversed by methysergide, indicating the participation of serotonin receptors in the antinociceptive effect of this toxin (Table S4) [304].

### 2.14. Not Known Targets

This topic focuses on toxins and crude venoms that exhibit antinociceptive action in animal models, but their pharmacological targets remain unidentified (Table S5). A compound named mygalin was identified from the spider *Acanthoscurria gomesiana* and presented an antinociceptive effect in an acetic acid-induced pain model. The authors suggest that, as it is an acyl-polyamine, it is possible that this molecule targets NMDA receptors [305]. In another study using the same pain model, the peptide named brachyin from *Brachypelma albopilosum* presented a similar effect [306].

Antinociceptive activities were also found in bees and wasps. Antinociceptive studies using the CFA-induced chronic arthritis and the capsaicin pain model demonstrated that the crude venom from *A. mellifera* has antinociceptive action. This effect was not inhibited by naloxone, a non-selective blocker of opioid receptors, indicating that the antinociceptive effect of the venom is not mediated by the opioid system [307,308,309].

In another study carried out with this species, the results obtained demonstrated that bee venom significantly increased the antinociceptive effect of ketoprofen and tramadol in the hot-plate model in mice, and the administration of intraperitoneal crude venom did not cause changes in motor activity [310]. In the formalin-induced inflammatory pain test, AmsBV, which comes from *Apis mellifera syriaca*, showed a decrease in nociceptive stimulation. The hot plate was used to measure the stimulus caused by the inflammatory agent. AmsBV reduced inflammatory cytokines, demonstrating an immunomodulatory effect [311].

The mastoparan Agelaia-MPI, originating from the wasp *Parachartergus fraternus*, demonstrated an important antinociceptive action. This effect was more pronounced in the hot-plate test than in the tail-flick test. These results suggest an interaction with non-opioid receptors by partial and reversible blockade of action potential amplitude, possibly in Nav channels. Another possibility would be an interaction with sodium channels, as the peptide was able to partially block the action potential in electrophysiological experiments conducted on the sciatic nerve. Further studies are necessary to establish a relationship between the compound and potential targets. Similar results were found in fractions of *Pseudopolybia vespiceps* venom [312,313].

The ant toxin named myrmexin found in *Pseudomyrmex triplarinus* and the crude venom of *Dinoponera quadriceps* present antinociceptive properties. Pain models using mechanical, thermal, and chemical stimuli have shown their effects in reducing nociception [314,315,316]. Poneratoxin (PoTX), which was isolated from *Paraponera clavata*, presented similar effects. Its exact pharmacological target has not been found, but it is thought to work on non-opioid receptors [317].

Among the scorpions, the species *B. martensii* stands out, presenting six different toxins, known as BmK IT-AP, BmK IT AP3, BmK AGAP-SYPU1, BmK AGAP-SYPU2, BmK AngP1, and BmK dITAP3, which induce antinociceptive effects. These toxins did not show toxic effects in blow-fly larvae or mammals; however, these compounds were found to induce dependence when compared to morphine, heroin, and aspirin [318,319,320,321,322,323]. The toxin leptucin, derived from the venom of *Hemiscorpius lepturus*, exhibited acute toxic effects and possibly interacted with opioid receptors, despite showing antinociceptive potential in thermal stimulus [324].

The centipede *S. s. mutilans* produces the SsmTX-I toxin, whose antinociceptive potential was evidenced using the formalin-induced pain test; pharmacological target tests were not conducted [325]. Toxins from cnidarians like *Stichodactyla mertensii*, *Stichodactyla gigantea*, *Pseudopterogorgia elisabethae*, and *Pelagia noctiluca* helped reduce pain in models of arthritis and inflammation caused by acetic acid without presenting toxic effects [326,327,328].

Toxins of seven species of *Conus* sp., including *C. striatus*, *C. coronatus*, *C. virgo*, and *C. frigidus*, showed antinociceptive effects in a formalin-induced pain model. *C. imperialis* toxin induced an antinociceptive effect in the nerve ligation-induced neuropathic pain model. The venom of *C. parvatus* presented a potent antinociceptive effect in mechanical stimulus, mediated by action in both the CNS and PNS, surpassing the effect of pentazocine [329,330,331]. On the other hand, this venom reduced the motor activity, which compromises the reliability of the results of nociception tests, which are based on motor behavioral responses. The crude venom of *C. virgo* and *C. striatus* induced an antinociceptive effect in inflammatory pain caused by formalin, carrageenan, and acetic acid, mainly in the acute phase with lower toxicity and tolerance [332,333].

Several bioactive compounds have been identified in the amphibian group. Bufotenin, an indolealkylamine extracted from toad venom, has demonstrated anti-inflammatory and antinociceptive effects caused by formalin. However, the compound showed toxicity in biological models. To alleviate the toxicity of the compound in the organism, the authors encapsulated it in liposomes, generating satisfactory results, since the desired effects were preserved [334,335,336,337].

The compound analgesin-HJ identified in *Hyla japonica* showed the ability to attenuate nociception associated with inflammation by modulating factors like TNF-α and IL-1β [338]. Peptide CI5, derived from *B. gargarizans*, manifested a reduction in abdominal contortion caused by acetic acid and a reduction in edema and inflammatory pain caused by carrageenan and formalin [339].

Neuropeptide B, derived from *B. gargarizans*, exhibited a transdermal antinociceptive effect in hot-plate and acetic acid pain models when associated with the antennapedia protein, which facilitates transmembrane permeability [340].

A comparative study using specimens of the snake *Bungarus fasciatus* from different regions of Vietnam demonstrated antinociceptive potential in the acetic acid test [341]. Studies using the *C. d. terrificus* venom fraction, crotoxin, demonstrated an antinociceptive effect mediated in the central nervous system, without reversal by blocking opioid and acetylcholine receptors [342,343].

Environmental factors, such as diet and geographic variations, significantly influence the potency and composition of venoms, as observed in comparative studies. This variability highlights the importance of identifying isolated molecules to ensure the reproducibility of pharmacological effects.

## 3. Trends and Challenges

The development of novel antinociceptive drugs encounters significant translational challenges hindering the progression of promising candidates from preclinical research to clinical application. Efforts to mitigate these challenges include a focus on new pharmaceutical agents that interact with receptors distinct from those currently available on the market, such as VGCs, TRPs, and ASICs, as well as consideration of the discrepancies observed between biological models utilizing rodents and human physiology. This highlights the need to develop new techniques to investigate specific pathways of action for pain treatment [344,345,346]. Alternatives have been explored to solve this problem, such as the use of biomarkers and 3D models of nociceptors to facilitate the understanding of the trajectory and effectiveness of new compounds [347,348].

The clinical success of venom-derived analgesics is underscored by the high affinity and specificity of these toxins for ion channels, exemplified by Ziconotide (Prialt^®^. TerSera Therapeutics LLC, Deerfield, IL, USA)—a synthetic ω-conotoxin MVIIA that targets N-type voltage-gated calcium channels (Cav2.2) to provide relief for refractory pain without opioid-related risks—and Tetrodotoxin (TTX/Halneuron^®^. Dogwood Therapeutics Inc., Alpharetta, GA, USA), which is utilized in palliative care. These molecules, shaped by millions of years of evolutionary refinement, achieve a level of molecular precision that traditional small-molecule drugs rarely attain, establishing venoms as essential “biological blueprints” for developing biopharmaceuticals that effectively bypass conventional opioid pathways [23,28].

There are still several challenges facing clinical candidates. A major challenge is the narrow therapeutic index; for instance, many Nav1.7 or Nav1.8 blockers derived from spider and scorpion toxins show remarkable efficacy in rodent models but fail in humans due to off-target effects on the cardiovascular or neuromuscular systems. Furthermore, the delivery remains a bottleneck. While Ziconotide requires invasive intrathecal administration to reach its CNS targets, many newer candidates (such as modified ω-conotoxins) have failed Phase II trials because they could not achieve stable therapeutic concentrations in human plasma due to rapid proteolytic degradation. The “translational gap”—where molecules perform well in reflex-based animal pain models but fail to address the complex, multidimensional nature of human chronic pain—remains the primary obstacle for candidates currently in the pipeline.

Furthermore, most antinociceptive toxins, in addition to the drugs mentioned above, have a molecular mass of less than 5 kilodaltons (kDa) and greater than 500 daltons (Da). According to Muttenthaler et al., approximately 75% of drugs marketed in 2019 were based on low-molecular-weight molecules, 20% were biopharmaceuticals, and 5% were peptides [349]. The work of Wang et al. and Craik et al. identified that the future of research on molecules with pharmaceutical potential will focus on peptides located between sizes of 500 Da and 5000 Da, since there is a gap in the development of peptide-based drugs with intermediate sizes, which may yield different results from those found in small and large molecules, such as greater specificity and precision with their pharmacological targets [350,351].

The area of study, called venomics, has achieved numerous advances in the study of animal toxins. By conducting an integrated analysis of the proteomes of the total venom, along with the transcriptomes of venom glands/ducts, it is possible to determine the fundamental molecules found and relate them to potential therapeutics [352].

Despite advances in “venomics”, technical limitations persist, especially in the detection of low-abundance toxins and in the characterization of complex post-translational modifications. The integration of transcriptomic and proteomic data is essential to overcome the gap between genetic identification and the biological functionality of toxins.

Intraspecific genetic variations, resulting from polymorphisms and differential gene regulation, can lead to the expression of isoforms with distinct pharmacological efficacies. Such genetic variability should be considered in the selection of lead molecules, as small structural alterations can significantly impact the interaction with molecular targets and the reproducibility of the observed analgesic effects. Although natural venom extraction faces scalability challenges and high operating costs, the economic viability of these drug candidates lies in the transition to recombinant production and chemical synthesis. These technologies allow for the biotechnological standardization necessary for the pharmaceutical market, mitigating biological variability and reducing mass production costs in the long term.

Furthermore, ethical considerations in the supply of venoms include compliance with animal welfare protocols and biodiversity access legislation. The transition to toxin production via recombinant technology or chemical synthesis, discussed in this review, is fundamental to reducing dependence on animal specimens and ensuring the sustainability of drug discovery.

## 4. Conclusions

To fully realize the clinical potential of toxins as biopharmaceuticals, research is increasingly focused on pharmacological optimization and innovative delivery systems. Strategies such as the development of synthetic analogs with enhanced stability and selectivity, as demonstrated with KIIIA and AC1 conotoxins, and the use of recombinant technology for scalable production of molecules like Phα1β and crotamine are critical steps toward therapeutic viability. Furthermore, to overcome the limitations of invasive administration—typically associated with intrathecal delivery—new routes are being explored, including intranasal and topical applications that have shown promising antinociceptive effects in preclinical models. The integration of nanotechnology also plays a pivotal role; for instance, the association of toxins with micelles or liposomes has proven effective in protecting these peptides from degradation, facilitating tissue permeation, and significantly reducing motor or systemic side effects. These multifaceted efforts to refine “biological blueprints” through bioengineering and nanotechnology are essential to bridging the translational gap and establishing toxins as the next generation of safe, non-opioid analgesics.

In summary, this review highlighted a wide range of animal venoms and toxins with potential for pain relief, most of which are intermediate-mass peptides and interact with voltage-gated ion channels. Nonetheless, the advantages and limitations in the development of new drugs, such as translatability between animal models and humans, advances in venom study technology, and synthetic peptide production, present promising solutions to bridge the gaps in the production of new drugs.

## 5. Materials and Methods

To begin this review, we asked the following questions: How many venom molecules and toxins with antinociceptive effects have been identified to date? And which receptors do they act on? Once these questions were posed, we began the process of searching for and selecting relevant articles.

**Figure 3:**
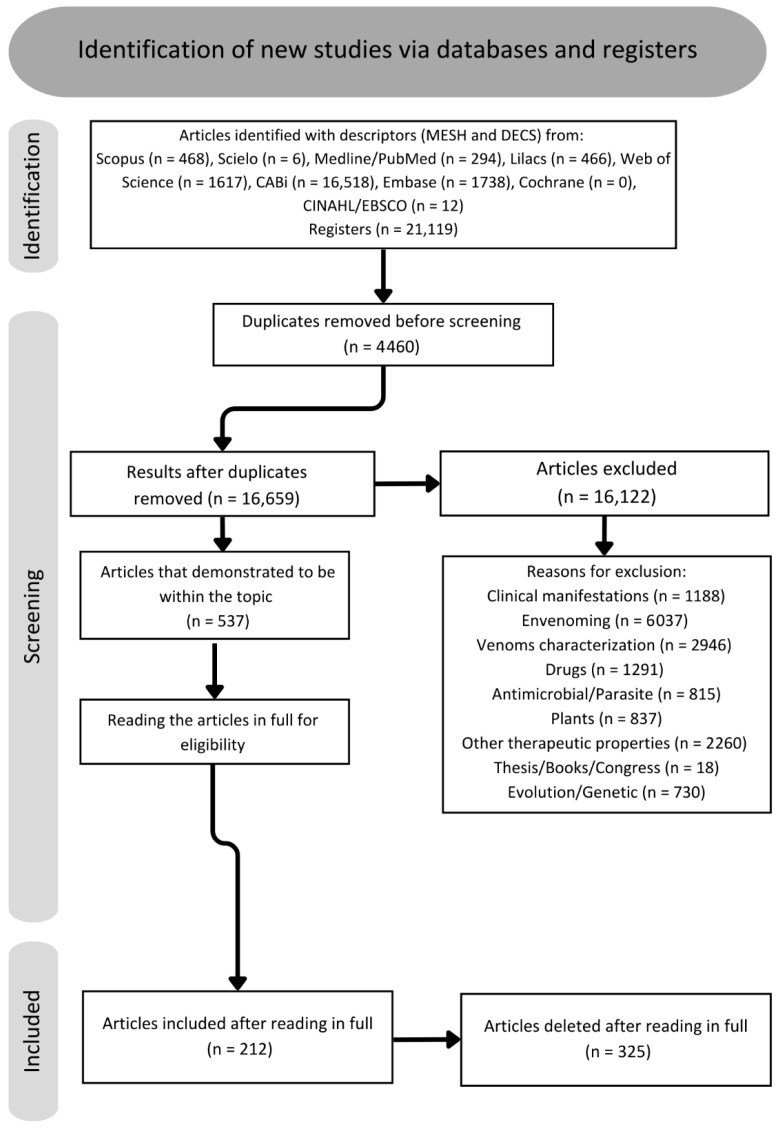
Flowchart about screening of articles.

The bibliographic search was conducted in January 2025, covering the period from 1960 to 2025. We used descriptors obtained from the MeSH and DECS platforms and performed searches in both Latin American databases (SciELO and LILACS) and international databases (Scopus, Web of Science, CABI, Cochrane, CINAHL, Embase, and Medline/PubMed) (Figure 3). The descriptor lists are in the Supplementary Material.

We then selected eligible articles based on the inclusion and exclusion criteria specified for this study, which included:

- Elimination of duplicates.
- Elimination of articles that did not address the target topic.
- Inclusion of original research articles and reviews that added new insights.
- Inclusion of articles on molecular characterization.
- Inclusion of experiments with positive and consolidated data.

When the title and abstract were aligned with the theme, the full text was read using the data extraction method. The flowchart below represents the criteria used to exclude and include articles (Figure 3). Furthermore, for each article that met the inclusion and data extraction criteria, we performed additional reading and selection of references within the article itself to delve into the subject and find new references on the study topic, including previous review articles on analgesia.

The information considered in each study included potentially active and antinociceptive molecules present in each animal group, their molecular size, their mechanism of action on related nociceptors, and properties of pharmacokinetic and pharmacodynamic importance.

## Data Availability

The original contributions presented in this study are included in the article/supplementary material. Further inquiries can be directed to the corresponding author.

## Supplementary Materials

The following supporting information can be downloaded at https://www.mdpi.com/article/10.3390/toxins18020069/s1, Table S1: Toxins that interact with voltage-gated channels to induce antinociception; Table S2: Toxins found to interact with opioid and cannabinoid receptors that cause antinociception; Table S3: Toxins found to interact with TRP receptors that cause antinociception; Table S4: Toxins found to interact with other receptors that cause antinociception; Table S5: Toxins with no known targets.

## Abbreviations

The following abbreviations are used in this manuscript:

VGCs	Voltage-gated channels
TRPs	Transient receptor potential
TNF	Tumor necrosis factor
DAMPs	Damage-associated molecular patterns
CB	Cannabinoid receptor
B	Bradykinin receptor
ASIC	Acid-sensitive ion channels
OP	Opioid receptor
P2X	Purinergic receptor
ADR	Adrenergic receptor
ADN	Adenosine receptor
5-TH	Serotonin receptor
Cav	Voltage-gated calcium channel
Nav	Voltage-gated sodium channel
Kv	Voltage-gated potassium channel
CCK	Cholecystokinin receptors
IGluRs	Ionotropic glutamate receptors
GABA	γ-Aminobutyric acid
kDa	Kilo daltons
CFA	Complete Freund’s adjuvant
CNS	Central nervous system
PNS	Peripheral nervous system
CCI	Chronic constriction injury
PBS	Phosphate-buffered saline
DDPCPX	Dipropylcyclopentylxanthine
ZZM241385	Potent selective adenosine A2A antagonist
PMA	Phorbol 12-myristate 13-acetate
NO	Nitric oxide
