# Supplementary material for: Exploring the Pain-Relieving Potential: Unveiling Antinociceptive Properties in Animal Venoms and Toxins
Source: Toxins (Basel). 2026 Jan 27;18(2):69. doi: 10.3390/toxins18020069 (PMC12945304; doi:10.3390/toxins18020069)
Supplement: Supplementary file 1 [file toxins-18-00069-s001.zip › toxins-4026348-supplementary.pdf]

toxins

MDPI

Supplementary materials: Exploring the Pain-Relieving Potential: Unveiling Antinociceptive Properties in Animal Venoms and Toxins

Davi Gomes Angstmam, Bruna Cristina Jeronimo, Joeliton dos Santos Cavalcante, Ana Flávia Marques Pereira, Cristiane Flora Villarreal, Daniel Carvalho Pimenta and Rui Seabra Ferreira Junior

Table S1. Toxins that interact with voltage-gated channels to induce antinociception.

Species	Group	Production	Toxin	Mass (kDa)	Targets	Administration	Models	Ref.
Agelenopsis aperta	Spider	Purification	ω-AGTX-IVA	5	Cav	Topical	Carrageenan	[75,76]
Bufo gargarizans	Amphibian	-	Bufalin	-	Nav	Intraperitoneal	Carrageenan<br>Formalin	[125]
Buthus martensii	Scorpion	Recombinant	AGAP	7.1	Nav	Intravenous	Formalin	[93,94]
Buthus martensii	Scorpion	Recombinant	W38G	-	Nav	Intravenous	Formalin	[93]
Buthus martensii	Scorpion	Purification	BmKIT2	-	Nav	Subcutaneous<br>Intraplantar	Carrageenan<br>Formalin	[95–97]
Buthus martensii	Scorpion	Purification	BmK AS	7.6	Nav	Intrathecal	Carrageenan<br>Formalin	[98–100]
Buthus martensii	Scorpion	Recombinant	BmK AGAP	-	Nav<br>Cav	Intravenous<br>Intrathecal	Formalin<br>Acetic acid<br>CCI	[101–104]
Buthus martensii	Scorpion	Purification<br>Recombinant	BmK AGP-SYPU-1	7.5	Nav	Intraperitoneal	Acetic acid	[105]
Buthus martensii	Scorpion	Recombinant	BmK DKK13	-	Nav	Intravenous	CCI<br>Mechanical allodynia	[106]
Buthus martensii	Scorpion	Recombinant	Makatoxin-3/ 3-R58A	9.4	Nav	Intraperitoneal	Formalin<br>Acetic acid<br>CFA	[111]
Buthus martensii	Scorpion	Recombinant	BmK AngM1	7.0	Nav<br>Kv	Intravenous	Acetic acid	[107]
Buthus martensii	Scorpion	Recombinant	Syb-prII-1	-	Nav	-	CCI	[108]
Buthus martensii	Scorpion	Purification	ANEPtoxin	-	Nav	Intravenous	Acetic acid	[109]
Buthus martensii	Scorpion	Recombinant	DKK-SP1;<br>DKK-SP2	10	Nav	Intravenous	CCI<br>Acetic acid	[110]
Buthus o. tanetanus	Scorpion	Purification	BotAF	7.4	Nav	Intrathecal	Formalin	[112]
Ceratogyrus darlingi	Spider	Synthetic	Cd1a	4.0	Nav<br>Cav	Intraplantar	OD1	[77]
Chilobranchys jingzhao	Spider	Synthetic	jingzhaotoxin-34	-	Nav	Intraperitoneal	Acetic acid<br>Formalin	[78]
Conus bullatus	Mollusk	Synthetic	Bu8	2.7	Cav	Intramuscular	Acetic acid	[43]
Conus catus	Mollusk	-	AM336	-	Cav	Intrathecal	CFA	[44]

Conus kinoshitai	Mollusk	Synthetic analogues	μ-conotoxin KIIIA	-	Nav	Intraperitoneal	Formalin	[45,46]
Conus magus	Mollusk	Ziconotide drug; peptide modified; Synthetic Recombinant	ω-conotoxin MVIIA (ziconotide)	2.6	Cav	Intracerebroventricular Intranasal Subcutaneous Intrathecal Intravenous	Formalin Acetic acid Nerve injury Neuropathic pain	[37–42]
Conus marmoreus	Mollusk	Synthetic Recombinant	μO-conotoxin MRVIB rMrVIB-His	-	Nav	Intradermal Subcutaneous Intrathecal	Incisional pain Neuropathic and Inflammatory pain Formalin CCI	[47–49]
Conus moncuri	Mollusk	Purification	ω-conotoxin MoVIA/ B	3.4	Cav	Intrathecal	Mechanical allodynia Pain nerve ligation	[50]
Conus regularis	Mollusk	Purification	RsXXIVA	-	Cav	Intraperitoneal	Formalin	[51]
Conus striatus	Mollusk	Synthetic	μ-conotoxin SO-3	2.6	Cav	Intracerebral Intrathecal	Formalin Light radiation	[52,53]
Conus tesselatus	Mollusk	Synthetic	μ-conotoxin TsIIIA	2.0	Nav	Intrathecal	Hot-plate	[54]
Cyriopagopus albostriatus	Spider	Purification	μ-TRTX-Ca1a; μ-TRTX-Ca2a	4.3;3.9	Nav	Intraperitoneal	Formalin	[68,69]
Cyriopagopus schioedtei	Spider	Synthetic	Cyriotoxina-1a (CyrTx-1a)	3.6	Nav	Intraplantar	Hot- plate PBS	[70]
Davus fasciatus	Spider	Purification	μ-TRTX-Df1a	4	Nav	Intraplantar	OD1	[83]
Grammostola porteri	Spider	Synthetic	GpTx-1;GpTx-71	-	Nav	Intrathecal Intracerebroventricular	CFA CCI Carrageenan Formalin OD1	[84–86]
Glovdius ussuriensis	Snake	Purification	Gln49-PLA2	13.8	Nav	Intraperitoneal	Hot-plate	[115,116]
Haplopelma lividum	Spider	Recombinant	μ-TRTX-Hl1a	4.3	Nav	intraperitoneal	Formalin Hot-plate Acetic acid	[87]
Heterometrus laoticus	Scorpion	Purification	Hetlaxin	3.6	Kv	Subcutaneous	Acetic acid Carrageenan	[114]
Heteropoda venatoria	Spider	Purification	Heteropodatoxin 3	-	Nav	Intraperitoneal	Formalin Acetic acid CFA Hot plate Spared nerve injury	[88]
Hottentotta franzwerneri	Scorpion	Synthetic	ω-Buthitoxin-Hf1a	1.1	Cav	Intraplantar	Post surgical pain model	[113]
Hyla annectans	Amphibian	Recombinant	Anntoxin	-	Nav	Intraperitoneal	Formalin Carrageenan	[127]
Hysterocrates gigas	Spider	-	SNX-482	-	Cav	Intrathecal	Spinal nerve ligation	[92]

Naja atra	Snake	Purification	μ-EPTX-Na1a	7.0	Nav	Intraperitoneal	Acetic acid<br>Formalin<br>CFA<br>Pain Nerve Ligation	[128]
Ornithoctonus hainana	Spider	Synthetic analogues	Hainantoxin-III/IV(HNTX-III); H4 [K0G1-P18K-A21L-V]	3.9	Nav	Intramuscular<br>Intraperitoneal	Formalin<br>Spinal Nerve Ligation<br>Acetic acid<br>CFA<br>Formalin	[71–74]
Ornithoctonus huwena	Spider	Purification	Huwentoxin-I	-	Cav	Intrathecal	Formalin	[89]
Ornithoctonus huwena	Spider	Recombinant	Huwentoxin-IV	-	Nav	-	OD1	[90]
Ornithoctonus huwena	Spider	Purification	Huwentoxin-XVI	4.4	Cav	Intramuscular<br>Intraperitoneal	Formalin<br>Hot-plate	[91]
Phlogiellus spp.	Spider	Synthetic analogues	Phlotoxin 1 (PhlTx1); D7A-PhlTx1	4.0	Nav	Intraplantar<br>Intrathecal	OD1<br>Formalin	[79,80]
Phoneutria nigriventer	Spider	Purification<br>Recombinant	Phα1β	6.0	Cav	Intrathecal<br>Intravenous	Acetic acid<br>Paclitaxel<br>CCI<br>Open-field<br>Formalin	[55–63]
Phoneutria nigriventer	Spider	Purification	Pntx3-3	6.3	Cav	Intrathecal<br>Intracerebroventricular	CFA<br>Fibromyalgia model<br>Hot Plate	[64,65]
Phoneutria nigriventer	Spider	Purification	PhTx3-4	8.4	Cav	Intrathecal	Formalin<br>Incisional pain<br>Acetic acid<br>NMDA	[66,67]
S. subspinipes multilans	Centipede	Recombinant	μ-SLPTX-Ssm6a	-	Nav	Intraperitoneal	Formalin<br>Acetic acid<br>Thermal pain	[129]
Tetraodontidae	Fish	-	Tetrodotoxin	-	Nav	Subcutaneous	Paclitaxel<br>Formalin<br>Carrageenan<br>Partial nerve ligation	[118–124]
Trachycephalus typhonius	Amphibian	Synthetic<br>Recombinant	Tt7	6.5	Nav	Intraperitoneal	Hot-plate	[126]
Thrixopelma pruriens	Spider	Purification<br>Recombinant	ProTxII/III	3.8	Nav	Intraplantar<br>Intraperitoneal<br>Intrathecal	OD1 induced<br>Formalin<br>Hargreaves	[81,82]

Cav: Voltage gated Calcium channel; Nav: Voltage gated Sodium channel; Kv: Voltage gated Potassium channel; CFA: Complete Freund's adjuvant; CCI: chronic constriction injury; PBS: Phosphate-buffered saline

Table S2. Toxins found to interact with opioid and cannabinoid receptors that cause antinociception.

Species	Group	Production	Toxin	Mass (kDa)	Targets	Administration	Models	Ref.
Apis mellífera	Honeybee	Purification	Adolapin	11	Opioid	Intraperitoneal Intraplantar	Acetic acid Prostaglandin	[136]
Bufo gargarizans	Amphibian	Synthesis	Bufalin	-	Opioid Cannabinoid (CB2)	Intraperitoneal	Carrageenan Acetic acid Formalin Hot Plate	[166,167]
Conus textile	Mollusk	Purification	3Tex/2	-	Cannabinoid (CB1 and CB2)	Intraperitoneal Intrathecal	Formalin	[161]
Crotalus d. terrificus	Snake	Purification Recombinant	Crotamine	4,8	Opioid	Intraperitoneal Subcutaneous Intraplantar	Hot-plate Acetic acid	[151,152]
Crotalus d. terrificus	Snake	Purification Synthesis	Crotalphine	1.5	κ-Opioid; Cannabinoid (CB2)	Oral Subcutaneous Intrathecal Intraplantar Intraperitoneal	Acetic acid Hot Plate Carrageenan	[169–171]
Micrurus lemniscatus	Snake	Purification	MIV	-	Opioid	Oral Intraperitoneal Subcutaneous	Acetic acid Formalin	[155]
Naja naja atra	Snake	Purification	Najanalgesin	6.7	Opioid	Intraperitoneal Intrathecal	Hot-plate Acetic acid	[153.154]
Ophiophagus hanna	Snake	Purification	Hannalgesin	-	Opioid	Intraperitoneal Oral Intracerebroventricular	Hot-plate	[144]
Phoneutria nigriventer	Spider	Purification	δ-Ctenitoxin-Pn1a	-	μ and δ-Opioid; Cannabinoid (CB1)	Intrathecal Intraplantar	Carrageenan Prostaglandin	[165]
Phoneutria nigriventer	Spider	Purification	PnPP-19	-	μ and δ-Opioid; Cannabinoid (CB1)	Subcutaneous Intrarplantar Intracerebroventricular	Prostaglandin	[163,164]
Phyllomedusa sp	Amphibian	Purification Synthesis	Deltorphins	-	δ -Opioid	Intracerebroventricular	Tail-flick	[142,143]
Phyllomedusa bicolor	Amphibian	Purification Synthesis	Dermorphins	-	μ -Opioid	Intracerebroventricular Intraperitoneal	Hote plate Tail-flick	[140,141]

Table S3. Toxins found to interact with TRP receptors that cause antinociception.

Species	Group	Production	Toxin	Mass (kDa)	Targets	Administration	Models	Ref.
Buthus martensii	Scorpion	Recombinant Purification	BmK AGAP	7.1	TRPV1	Intrathecal	CCI	[101,197]

Grammostola rosea	Spider	Synthesis<br>Recombinant	GsMTx-4	4	TRPC1,<br>TRPC6,<br>TRPV4	Intradermal<br>Intraperitoneal	Carrageenan<br>Paclitaxel<br>Hindpaw Thermal Injury<br>Acid acetic<br>Hot-plate<br>Spinal Nerve Ligation	[195,196]
Heteractis crispa	Cnidarian	Recombinant	APHC-1<br>APHC-3	6	TRPV1	Intramuscular<br>Intravenous	Capsaicin<br>Formalin<br>Hot-plate<br>CFA<br>Acetic acid	[183,184]
Metridium senile	Cnidarian	Recombinant	Ms9a-1	3.6	TRPA1	Intravenous<br>Intraplantar	Allyl Isothiocynate induced<br>CFA model<br>Capsaicin test	[185,186]
Phoneutria nigriventer	Spider	Purification<br>Recombinant	Pntx3-5	5	TRPV1	Intrathecal<br>Intradermal	Hot-plate<br>Postoperative pain model<br>Partial nerve ligation model<br>Cancer (Melanoma) pain model	[188,189]
Phoneutria nigriventer	Spider	Purification	Phα1β	6	TRPV1;<br>TRPA1	Intrathecal<br>Intradermal<br>Intraplantar	Capsaicin<br>CCI	[190–194]
Urticina eques	Cnidarian	Recombinant	Ueq 12-1	4.8	TRPA1	Intraplantar<br>Intravenous	Allyl Isothiocynate<br>CFA	[187]

CFA: Complete Freund's adjuvant; CCI: chronic constriction injury.

Table S4. Toxins found to interact with other receptors that cause antinociception.

Species	Group	Production	Toxin	Mass (kDa)	Targets	Administration	Models	Ref.
Apis mellifera	Honeybee	Diluted venom	-	-	α2-Adrenergic	Subcutaneous	Paclitaxel<br>Acetic acid<br>CCI model<br>Collagen-Induced Arthritis<br>Oxaliplatin<br>Formalin<br>Acetic acid	[265,279–281]
Apis mellifera	Honeybee	Diluted venom	-	-	NMDA	Subcutaneous	CCI	[265,266]
Bufo gargarizans	Amphibian	Purification	Bufalin	-	P2X7	Intraperitoneal	Hot-plate<br>Acetic acid<br>CCI	[274]
Bufo bufo gargarizans	Amphibian	-	Cinobufagin	-	nAChRs	Intraperitoneal<br>Intrathecal	Hot-plate<br>Acetic acid Formalin	[237]
Bunodosoma cangicum	Cnidarian	Synthetic	Bunodosin 391	-	Serotonin	Intraplantar	Carrageenan	[308]
Conus achantinus	Mollusk	Synthetic	Conotoxin-Ac-1	1.9	NMDA	Lateral injection	Hot-plate<br>Tail-flick	[258]
Conus aulicus	Mollusk	Synthetic	α-Conotoxin AuIB	-	GABA	Intraperitoneal	Acetic acid<br>Pain Nerve Ligation	[250]

Conus bullatus	Mollusk	Synthetic	analogue BuIA	1.6	nAChRs	Intrathecal	Paclitaxel<br>Hot-plate	[222]
Conus generalis	Mollusk	Synthetic	αO-conotoxin GeXIVA	-	nAChRs	Intramuscular	Oxalapatin	[223]
Conus geographus	Mollusk	Synthetic	Conantokin G<br>Conantokin T	-	NMDA	Intrathecal	Formalin<br>Nerve injury<br>CFA<br>Hot-plate<br>Acetic acid	[259,260]
Conus litteratus	Mollusk	Synthetic	lt14a	1.3	nAChRs	intraperitoneal	Hot-plate	[224]
Conus lividus	Mollusk	Synthetic	α -conotoxin Lv1d	1.7	nAChRs	Intrathecal	Hot-plate<br>Formalin	[225]
Conus marmoreus	Mollusk	Synthetic	α -conotoxin Mr1.1	1.7	nAChRs	Intraplantar	Carrageenan	[226]
Conus regius	Mollusk	Synthetic	α-conotoxin RgIA	-	nAChRs	Intramuscular	Oxaliplatin<br>Cold plate	[227,228]
Conus victoriae	Mollusk	Synthetic	α-conotoxin Vc1.1	1.8	GABA; nAChRs	Intramuscular	Spinal Nerve Ligation<br>Blister Induction<br>Antidromic Electrical Stimulation of Sciatic Nerve	[229,230]
Dendroaspis polylepis	Snake	Synthetic	Mambalgin I; II; III	4.6	ASICs	Intrathecal	Carrageenan	[209,210]
Epipedobates tricolor	Amphibian	Synthetic	Epibatidine	-	nACh Rs	Subcutaneous<br>Intrathecal	Acute thermal nociception<br>CFA<br>Partial Nerve Ligation<br>Spinal nerve<br>Acute and Chronic tolerance	[216–221]
Geolycosa sp.	Spider	Recombinant	Purotoxin-1,-2		P2X3	Intraplantar	Formalin<br>Capsaicin<br>Carrageenan<br>CFA	[272,273]
Heteractis crispa	Cnidarian	Purification	Hcr1b-1, 2, 3, 4	4-4.5	ASIC1a; ASIC3	Intraperitoneal	Acetic acid	[206,207]
Hyla caerulea	Amphibian	Purification	Caeruleín	4.6	CCK	Subcutaneous<br>Intrathecal	Tail-flick<br>Hot-plate<br>Writing	[302,303]
Naja naja atra	Snake	Purification	CNT	-	Adenosine ($A_1$and A_2)	Intraperitoneal	Spinal Pain Ligation<br>Hot-plate<br>DPCPX<br>ZM241385	[293]
Naja naja atra	Snake	-	Cobrotoxin (CT)	-	nAChRs	Intracerebroventricular	Atropine<br>Methylatropine<br>Hemicholinium-3 (HC-3)	[231]

Naja naja kaouthia	Snake	-	Cobratoxin (CTX)	-	nAChRs	Intracerebroventricular<br>Intrathecal	para-Chlorophenylalanine<br>CFA<br>Cold plate<br>Formalin<br>Partial Sciatic Nerve ligation<br>Cancer-induced bone pain	[232–236]
Phoneutria nigriventer	Spider	Purification	PhKv (PhTx3-1)	-	nAChRs	Intrathecal	Carrageenan<br>CCI<br>Capsaicin<br>Acetylcholinesterase	[238]
Phoneutria nigriventer	Spider	Purification	Pntx(5-5)	4.5	NMDA	Intrathecal	NMDA-induced spontaneous nociception	[263]
Polybia occidentalis	Wasp	Purification	Thr6-BK	1	Bradykinin (B2)	Intracerebroventricual	Hot-plate<br>Tail-flick	[286]
Trichonephila clavata	Spider	Synthetic	JST	0.565	AMPA	Intrathecal	Carrageenan<br>Formalin<br>Thermal Hyperalgesia<br>Mechanical Allodynia	[261,262]
Tityus serrulatus	Scorpion	Purification	TsNTxP	-	NMDA	Intrathecal	CCI<br>Paclitaxel<br>Capsaicin	[264]
Urticina grebelnyi	Cnidarian	Recombinant	UGR9a-1	3	ASIC3	-	CFA<br>Acetic acid	[208]

Non peptide; DPCPX: Dipropylcyclopentylxanthine; ZM241385: potent selective adenosine A2A antagonist; CFA: Complete Freund's adjuvant; CCI: chronic constriction injury.

Table S5. Toxins found to interact no known receptors that cause antinociception.

Species	Group	Production	Toxin	Mass (kDa)	Targets	Administration	Models	Ref.
Acanthoscurria gomesiana	Spider	Synthetic	Mygalin	-	-	Intracerebroventricular	Hot-plate<br>Acetone	[309]
Apis mellifera	Honeybee	Crude venom	-	-	-	Subcutaneous<br>Intraperitoneal	Chronic post-ischemia pain<br>CFA<br>Hargreave's<br>Formalin	[311–314]
Apis mellifera syriaca	Honeybee	Crude venom	-	-	-	Intraperitoneal	Formalin<br>Hot-plate	[315]
Brachypelma albopilosum	Spider	Purification	Brachyin	4.9	-	Intraperitoneal	Formalin test	[310]
Bufo gargarizans	Amphibian	Synthetic	Bufotenine	-	*	Intraperitoneal	Formalin<br>Hot-plate	[338–340]
Bufo gargarizans	Amphibian	Synthetic	CI5	-	-	Intraperitoneal	Carrageenan<br>Formalin	[343]

							Acetic acid	
Bufo gargarizans	Amphibian	Purification	Peptide B	5.5	-	Transdérmic	Hot-plate<br>Acetic acid	[344]
Bungarus fasciatus	Snake	Purification	BF-4VS	-	-	Intraperitoneal	Acetic acid	[345]
Buthus martensii	Scorpion	Purification	BmK IT-AP	-	-	Intravenous	Acetic acid	[322]
Buthus martensii	Scorpion	Purification	BmK AGAP-SYPU1	-	-	Intraperitoneal	Acetic acid	[324]
Buthus martensii	Scorpion	Purification	BmK AGAP-SYPU2	-	-	Intravenous	Acetic acid	[325,326]
Buthus martensii	Scorpion	Purification	BmK AngP1	8.1	-	Intravenous	Acetic acid	[327]
Buthus martensii	Scorpion	Purification	BmK dITAP3	6.5	-	Intravenous	Acetic acid	[323]
Conus coronatus	Mollusk	Purification	-	<5	-	Intracranial	Formalin Hot-plate	[333]
Conus frigidus	Mollusk	Purification	-	<5	-	Intracranial	Formalin Hot-plate	[333]
Conus imperialis	Mollusk	Synthetic	Im10A	-	-	Intramuscular	Partial Nerve Ligation	[334]
Conus parvatus	Mollusk	Crude venom	-	-	-	Intraperitoneal	Tail-flick<br>Rota-rod	[335]
Conus striatus	Mollusk	Recombinant	μ-conotoxin S24a	-	-	Intrathecal	Hot-plate<br>Formalin	[336]
Conus virgo	Mollusk	Crude venom	-	-	-	Intraperitoneal	Acetic acid Carrageenan	[337]
Crotalus durissus terrificus	Snake	Purification	Crotoxin	24	-	Intraperitoneal<br>Intracerebroventricular	Hot plate<br>Acetic acid<br>Formalin<br>Hargreaves	[346,347]
Dinoponera quadriceps	Ant	Crude venom	-	-	-	Intravenous	Formalin<br>Hot-plate<br>Wound model	[319,320]
Hemiscorpius lepturus	Scorpion	Synthetic	Lepucitin	-	-	Intraperitoneal	Hot-plate<br>Tail-flick	[328]
Hyla japonica	Amphibian	Synthetic	Analgesin	-	-	Intraperitoneal	Acetic acid<br>Formalin<br>Hot-plate	[342]
Parachartergus fraternus	Wasp	Synthetic	Agelaia-MPI	1.5	-	Intracerebroventricular	Hot-plate<br>Tail-flick<br>Rota-rod	[317]
Paraponera clavata	Ant	-	Poneratoxin (PoTX)	2.9	-	Intracerebroventricular	Chloral hydrate	[321]
Pelagia noctiluca	Cnidarian	Crude venom	-	-	-	Intraperitoneal	Acetic acid	[332]
Pseudomyrmex triplarinus	Ant	Purification	Myrmexin	7	-	Subcutaneous	Carrageenan	[318]
Pseudopolybia vespiceps testacea	Wasp	Purification	-	3	-	intracerebroventricular	Tail-flick<br>Hot-plate	[316]

Pseudopterogorgia elisabethae	Cnidarian	Purification	Pseudopterosin E	-	-	Intraperitoneal Subcutaneous	PMA	[331]
Rhinella jimi	Amphibian	Purification	Telocinobufagin	-	-	Oral Intraperitoneal	Acetic acid Formalin Hot-plate	[341]
Scolopendra subspinipes mutilans	Centipede	Synthetic	SsmTX-I	4,1	-	Intraperitoneal	Formalin	[329]
Stichodactyla mertensii	Cnidarian	Crude venom	-	-	-	Intraperitoneal Intramuscular	Tail-flick Hot-plate	[330]
Stichodactyla gigantea	Cnidarian	Crude venom	-	-	-	Intraperitoneal Intramuscular	Tail-flick Hot-plate	[330]

*Non peptide; CFA: Complete Freund's adjuvant; PMA: Phorbol 12-Myristate 13-Acetate

Descriptors used to perform the literature review:

The list of descriptors used on Latin American platforms based on the Descriptors obtained from DECS was as follows: ((Peçonhas OR Venoms OR Ponzoñas OR Venenos de Animais) OR (Venenos de Anfíbios OR Amphibian Venoms OR Venenos de Anfibios OR Venenos de Rã OR Venenos de Salamandra OR Venenos de Sapo) OR (Venenos de Artrópodes OR Arthropod Venoms OR Venenos de Artrópodos OR Toxina de Aracnídeo OR Toxinas de Aracnídeos OR Veneno de Artrópodes OR Veneno de Himenóptero OR Veneno de Himenópteros OR Veneno de Inseto OR Veneno de Insetos OR Venenos de Aracnídeos OR Venenos de Himenópteros OR Venenos de Insetos) OR (Venenos de Serpentes OR Snake Venoms OR Venenos de Serpiente OR Toxina de Serpente OR Toxinas de Serpente OR Venenos de Ofídios) OR (Venenos de Peixe OR Fish Venoms OR Venenos de los Peces OR Veneno de Peixe) OR (Venenos de Vespas OR Wasp Venoms OR Venenos de Avispas OR Veneno de Vespa OR Venenos de Vespa OR Venenos de Vespídeos) OR (Venenos de Moluscos OR Mollusk Venoms OR Venenos de Moluscos OR Veneno de Molusco OR Venenos de Caramujo OR Venenos de Conus OR Venenos de Octopus OR Venenos de Polvo) OR (Venenos de Cnidários OR Cnidarian Venoms OR Venenos de Cnidarios OR Venenos de Anêmona-do-Mar OR Venenos de Chironex OR Venenos de Cnidaria OR Venenos de Medusa OR Venenos de Nematocistos) OR (Venenos de Abelha OR Bee Venoms OR Venenos de Abeja OR Apitoxina OR Veneno de Abelha OR Veneno de Abelha Melífera OR Venenos de Abelhas Melíferas OR Venenos de Apis) OR (Venenos de Formiga OR

Ant Venoms OR Venenos de Hormiga OR Venenos de Formicoidea) OR (Venenos de Víboras OR Viper Venoms OR Venenos de Víboras OR Toxina de Víbora OR Toxinas de Víboras OR Toxinas de Víperídeos OR Toxinas de Víperíneos OR Veneno da Daboia russelii OR Veneno da Víbora Cornuda Egípcia OR Veneno da Víbora de Areia Egípcia OR Veneno da Víbora de Russell OR Veneno da Víbora-de-Russell OR Veneno de Cerastes viper OR Veneno de Daboia russelii OR Veneno de Víbora OR Veneno de Víbora de Russell OR Veneno de Víbora-de-Russell OR Venenos da Daboia russelii OR Venenos da Víbora de Russell OR Venenos da Víbora-de-Russell OR Venenos de Daboia russelii OR Venenos de Viperidae OR Venenos de Viperídeos OR Venenos de Víbora OR Venenos de Víbora de Russell OR Venenos de Víbora-de-Russell OR Viperotoxina) OR (Venenos Elapídicos OR Elapid Venoms OR Venenos Elapídicos OR Veneno Elapídico OR Veneno Hidrofídico OR Veneno de Cobra Real OR Veneno de Elapidae OR Veneno de Naja OR Veneno de Ophiophagus hannah OR Veneno de Serpentes Marinhas OR Venenos Hidrofídicos OR Venenos de Elapidae OR Venenos de Naja OR Venenos de Serpentes Marinhas) OR (Venenos de Serpentes OR Snake Venoms OR Venenos de Serpiente OR Toxina de Serpente OR Toxinas de Serpente OR Venenos de Ofídios) OR (Venenos de Crotalídeos OR Crotalid Venoms OR Venenos de Crotálidos OR Crotactina OR Crotalina OR Crotalotoxina OR Crotamina OR Veneno de Bothrops OR Veneno de Bothrops jararaca OR Veneno de Cobra Crotalina OR Veneno de Crotalinae OR Veneno de Crotalídeo OR Veneno de Crotalíneos OR Veneno de Serpente Crotalina OR Veneno de Víbora Crotalina OR Venenos de Cascavel OR Venenos de Crotalídeo) OR (Venenos de Aranha OR Spider Venoms OR Venenos de Araña OR Fator de Crescimento Insulina-Like OR Fator de Crescimento Insulina-Símile OR Fator de Crescimento Similar à Insulina OR Toxina da Tarântula OR Toxina de Aranha OR Toxina de Tarântula OR Toxinas das Aranhas OR Toxinas de Aranha OR Toxinas de Aranhas OR Toxinas de Tarântulas OR Veneno da Tarântula OR Veneno de Aranha OR Veneno de Aranhas OR Veneno de Tarântula OR Veneno de Tarântulas OR Venenos das Tarântulas OR Venenos de Araneídeos OR Venenos de Aranhas OR Venenos de Tarântula OR Venenos de Tarântulas) OR (Venenos de Escorpião OR Scorpion Venoms OR Venenos de Escorpión OR Peptídeo de Veneno de Escorpião OR Toxina de Escorpião OR Toxinas de Escorpião OR Veneno de Tityus serrulatus)) AND (Analgesia).

The list of descriptors used in the search for international platforms based on the MESH platform were: ((Venoms OR Venom) OR (“Amphibian Venoms” OR “Venoms, Amphibian” OR “Amphibian Venom” OR “Venom, Amphibian” OR “Frog Venoms” OR “Venoms, Frog” OR “Frog Venom” OR “Venom, Frog” OR “Toad Venom” OR “Venom, Toad” OR “Toad Venoms” OR “Venoms, Toad”) OR (“Arthropod Venoms” OR “Venoms, Arthropod” OR “Arthropod Venom” OR “Venom, Arthropod” OR “Hymenoptera Venoms” OR “Venoms, Hymenoptera” OR “Hymenoptera Venom” OR “Venom, Hymenoptera” OR “Arachnid Venoms” OR “Venoms, Arachnid” OR “Arachnid Venom” OR “Venom, Arachnid” OR “Insect Venoms” OR “Venoms, Insect” OR “Insect Venom” OR “Venom, Insect” OR “Arachnid Toxins” OR “Toxins, Arachnid” OR “Arachnid Toxin” OR “Toxin, Arachnid”) OR (“Snake Venoms” OR “Venoms, Snake” OR “Snake Venom” OR “Venom, Snake” OR “Snake Toxins” OR “Toxins, Snake” OR “Snake Toxin” OR “Toxin, Snake”) OR (“Elapid Venoms” OR “Venoms, Elapid” OR “Elapidae Venom” OR “Venom, Elapidae” OR “Elapid Venom” OR “Venom, Elapid” OR “Elapidae Venoms” OR “Venoms, Elapidae” OR “King Cobra Venom” OR “Venom, King Cobra” OR “Ophiophagus hannah Venom” OR “Venom, Ophiophagus hannah” OR “Hydrophid Venoms” OR “Venoms, Hydrophid” OR “Sea Snake Venoms” OR “Venoms, Sea Snake” OR “Hydrophid Venom” OR “Venom, Hydrophid” OR “Sea Snake Venom” OR “Venom, Sea Snake” OR “Cobra Venoms” OR “Venoms, Cobra” OR “Naja Venoms” OR “Venoms, Naja” OR “Naja Venom” OR “Venom, Naja” OR “Cobra Venom” OR “Venom, Cobra”) OR (“Viper Venoms” OR “Venoms, Viper” OR “Viper Venom” OR “Venom, Viper” OR “Viperidae Venoms” OR “Venoms, Viperidae” OR “Egyptian Sand Viper Venom” OR “Viperotoxin” OR “Russells Viper Venoms” OR “Russells Viper Venoms” OR “Venoms, Russells Viper” OR “Viper Venoms, Russells” OR “Russells Viper Venom” OR “Russells Viper Venom” OR “Venom, Russells Viper” OR “Viper Venom, Russells” OR “Russell Viper Venom” OR “Venom, Russell Viper” OR “Viper Venom, Russell” OR “Russell Viper Venoms” OR “Venoms, Russell Viper” OR “Viper Venoms, Russell” OR “Cerastes Venoms” OR “Venoms, Cerastes” OR “Cerastes Venom” OR “Venom, Cerastes”) OR (“Fish Venoms” OR “Venoms, Fish” OR “Fish Venom” OR “Venom, Fish”) OR (“Fishes, Poisonous” OR “Poisonous Fishes”) OR (“Wasp Venoms” OR “Venoms, Wasp” OR “Vespid Venom” OR “Venom, Vespid” OR “Wasp Venom” OR “Venom, Wasp” OR “Vespa Venoms” OR “Venoms, Vespa” OR “Vespid Venoms” OR “Venoms, Vespid”) OR (“Mollusk Venoms” OR “Venoms, Mollusk” OR “Mollusk Venom” OR “Venom,

Mollusk" OR "Mollusc Venoms" OR "Venoms, Mollusc" OR "Octopus Venoms" OR "Venoms, Octopus" OR "Octopus Venom" OR "Venom, Octopus" OR "Snail Venom" OR "Venom, Snail" OR "Snail Venoms" OR "Venoms, Snail" OR "Conus Venoms" OR "Venoms, Conus" OR "Conus Venom" OR "Venom, Conus") OR ("Cnidarian Venoms" OR "Venoms, Cnidarian" OR "Cnidarian Venom" OR "Venom, Cnidarian" OR "Sea Anemone Venom" OR "Venom, Sea Anemone" OR "Sea Anemone Venoms" OR "Venoms, Sea Anemone" OR "Nematocyst Venoms" OR "Venoms, Nematocyst" OR "Portuguese Man-of-War Venom" OR "Portuguese Man of War Venom" OR "Venom, Portuguese Man-of-War" OR "Chironex Venoms" OR "Venoms, Chironex" OR "Chironex Venom" OR "Venom, Chironex" OR "Jellyfish Venoms" OR "Venoms, Jellyfish" OR "Jellyfish Venom" OR "Venom, Jellyfish") OR ("Bee Venoms" OR "Venoms, Bee" OR "Bee Venom" OR "Venom, Bee" OR "Apis Venoms" OR "Venoms, Apis" OR "Apitoxin" OR "Honeybee Venoms" OR "Venoms, Honeybee" OR "Honeybee Venom" OR "Venom, Honeybee") OR ("Ant Venoms" OR "Venoms, Ant" OR "Formicoidea Venoms" OR "Venoms, Formicoidea" OR "Ant Venom" OR "Venom, Ant" OR "Fire Ant Venoms" OR "Venoms, Fire Ant") OR ("Crotalid Venoms" OR "Venoms, Crotalid" OR "Pit Viper Venom" OR "Venom, Pit Viper" OR "Viper Venom, Pit" OR "Pit Viper Venoms" OR "Venoms, Pit Viper" OR "Crotalid Venom" OR "Venom, Crotalid" OR "Crotalin" OR "Crotaline Snake Venom" OR "Snake Venom, Crotaline" OR "Venom, Crotaline Snake" OR "Crotalotoxin" OR "Bothrops Venom" OR "Venom, Bothrops" OR "Bothrops jararaca Venom" OR "Venom, Bothrops jararaca" OR "Crotamin" OR "Rattlesnake Venoms" OR "Venoms, Rattlesnake" OR "Rattlesnake Venom" OR "Venom, Rattlesnake" OR "Crotactin") OR ("Spider Venoms" OR "Venoms, Spider" OR "Araneid Venoms" OR "Venoms, Araneid" OR "Spider Venom" OR "Venom, Spider" OR "Araneid Venom" OR "Venom, Araneid" OR "Tarantula Venoms" OR "Venoms, Tarantula" OR "Tarantula Venom" OR "Venom, Tarantula" OR "Tarantula Toxins" OR "Toxins, Tarantula" OR "Tarantula Toxin" OR "Toxin, Tarantula" OR "Spider Toxins" OR "Toxins, Spider" OR "Spider Toxin" OR "Toxin, Spider") OR ("Scorpion Venoms" OR "Scorpion Toxins" OR "Scorpion Toxin" OR "Toxin, Scorpion" OR "Scorpion Venom" OR "Venom, Scorpion" OR "Scorpion Venom Peptide" OR "Peptide, Scorpion Venom" OR "Venom Peptide, Scorpion" OR "beta-Scorpion Toxin" OR "Toxin, beta-Scorpion" OR "beta Scorpion Toxin" OR "gamma-

Scorpion Toxin" OR "gamma Scorpion Toxin" OR "Tityus serrulatus Venom" OR "Venom, Tityus serrulatus" OR "alpha-Scorpion Toxin" OR "Toxin, alpha-Scorpion" OR "alpha Scorpion Toxin")) AND (Analgesia OR Analgesias)
